# Effects of Different Polypropylene (PP)-Backbones in Aluminium Feedstock for Fused Filament Fabrication (FFF)

**DOI:** 10.3390/polym15143007

**Published:** 2023-07-11

**Authors:** Vahid Momeni, Zahra Shahroodi, Joamin Gonzalez-Gutierrez, Lukas Hentschel, Ivica Duretek, Stephan Schuschnigg, Christian Kukla, Clemens Holzer

**Affiliations:** 1 Polymer Processing, Montanuniversitaet Leoben, 8700 Leoben, Austria; 2 Functional Polymers Research Unit, Materials Research and Technology (MRT) Department, Luxembourg Institute of Science and Technology (LIST), L-4940 Hautcharage, Luxembourg; 3 Industrial Liaison Department, Montanuniversitaet Leoben, 8700 Leoben, Austria

**Keywords:** metal material extrusion, fused filament fabrication, aluminium feedstock, printability, binder system, rheological properties

## Abstract

The current study presents the effect of the backbone as an important binder component on the mechanical, rheological, and thermal properties of Aluminium (Al) alloy feedstocks. A thermoplastic elastomer (TPE) main binder component was blended with either polypropylene (PP), grafted-maleic anhydride-PP (PPMA), or grafted-maleic anhydride-PPwax (PPMAwax) plus PP, as the backbone. Differential scanning calorimetry (DSC) and thermogravimetric analysis (TGA) tests were performed to investigate the thermal properties of binder systems and feedstocks. Fourier-transform infrared (FTIR) spectroscopy was used to study the chemical interaction between the binder and the Al alloy. After making feedstock filaments, tensile tests, scanning electron microscopy (SEM), and fused filament fabrication (FFF) printing were done. The results showed that although the PP printability was acceptable, the best mechanical properties and printed quality can be achieved by PPMA. TGA test showed that all binder systems in the feedstocks could be removed completely around 500 °C. From FTIR, the possibility of chemical reactions between Al alloy particles and maleic anhydride groups on the grafted PP backbone could explain the better dispersion of the mixture and higher mechanical properties. Tensile strength in PP samples was 3.4 MPa which was improved 1.8 times using PPMA as the backbone.

## 1. Introduction

Shaping, debinding, and sintering (SDS) encompasses, among others, extrusion-based additive manufacturing, and powder injection molding (PIM) techniques [1]. The SDS process consists of four stages: material preparation (granules or filaments), shaping (injection molding or 3D printing), debinding, and sintering [2]. The feedstock, the raw material for this method, comprises a mixture of metal or ceramic powder and a binder system. Binders are multi-component combinations of several polymers and additives that play a significant role in the SDS processing of components [3]. It has been shown that just one component modification in the binder system may significantly alter feedstock characteristics [4,5]. After the preparation of the feedstock, green parts with the desired shape are produced by injection molding or 3D printing [6]. During the debinding, all components of the binder systems are extracted from the green part gradually using different techniques and without causing any defect [7]. Sintering, the final step, is done in the furnace by heating parts to temperatures lower than the melting point of the metal or ceramic used to achieve a nearly complete density part with high mechanical properties [8,9].

During the injection step in the PIM process, an expensive and unique mold made of durable materials is needed due to high-pressure levels and heating cycles. PIM is a cost-effective manufacturing technique to produce vast quantities of complex-shaped components [10,11]. However, the high cost of the mold makes this approach less viable for small quantities. Therefore, various approaches using additive manufacturing technologies for feedstocks have been developed to manufacture green parts as part of the SDS process [12]. One of these techniques is the extrusion-based AM method called the fused filament fabrication (FFF) process [13].

In contrast to PIM, where the feedstock is often supplied as granules [14], feedstock in the form of filament is required. FFF equipment installation expenses are significantly less than those for PIM equipment [15]. Although the FFF technique produces an almost limitless range of geometries without expensive molds, the quality of final parts should be improved compared to PIM parts by adjusting the process parameters and designing proper binder formulations. However, processing SDS feedstocks is significantly more complex than printing polymers generally used for FFF since they include a high amount of ceramic or metal powder [16]. In addition to appropriate flow properties, FFF filaments must be strong and flexible enough to be easily spooled, de-spooled, and extruded in the printing head, which is quite challenging when there is a high-volume fraction of powder [17]. Consequently, only a few commercial feedstocks are available [18,19,20]. However, many researchers have used FFF for different metals such as hard metals [21], Fe [22,23], steel [24,25], copper (Cu) [23], Ti-Al-V [26,27], W-Cr [28], Ni-Ti [29], Mg-Al-Zn [30] along with various binder formulations.

The binder systems generally consist of the main component and a backbone component to which various additives like dispersants, plasticizers, and surfactants are added [31]. A thermoplastic polymer with a good combination of stiffness and flexibility is often the main component [32]. Backbones help in shape retention of the green part during the debinding step when the main binder component is removed [33]. Improving the flowability of the feedstock and spreading the powder throughout the polymer matrix to avoid agglomeration could be done by plasticizers and surfactants [34]. It has been demonstrated that a binder system consisting of a thermoplastic elastomer (TPE) as a major component could be utilized successfully for the FFF process because it can provide high flexibility and extrusion reliability for the filaments [35]. In addition, the backbone’s type and concentrations determine the binder system’s debinding performance. Therefore, choosing a suitable backbone is crucial for developing a proper feedstock for the SDS process.

In addition, Hausnerova [36] reviewed the different binder systems for PIM feedstocks. It is suggested that polypropylene (PP) provides higher mechanical properties than other backbone polymers. In the study of Gökmen and Türker [37], the rheological behavior of the Inconel 625 superalloy feedstock with PP as the backbone was analyzed. They found that using PP as the backbone has the highest impact on the rheology behavior compared to the other binder constituents and improves the injection molding process. This study showed that binder content (especially backbone polymer) strongly influences the rheological properties of feedstocks.

Some studies have been conducted to develop binder systems for the FFF of different metals and ceramics. In the work of Cano et al. [38], a suitable binder formulation was developed for the zirconia powder. The feedstock with the binder system consisting of styrene-ethylene/butylene-styrene (SEBS)/paraffin wax (PW) was printed successfully without any visible defects in printing and after debinding. According to Gorjan et al. [39], ethylene vinyl acetate (EVA) with different contents of stearic acid was successfully applied for the FFF process of alumina. A single-component binder system containing low-density polyethylene (LDPE) was also utilized for the 316L stainless steel powder [40]. The mechanical test findings indicate that the FFF printed and sintered material is equivalent to MIM products. In another study, Nötzel and Hanemann [41] used Polyvinyl butyral (PVB) as the backbone and Polyethyleneglycol (PEG 4000) as the plasticizer, which is soluble in water. The best attainable sinter densities for the complex alumina parts like small gear wheels were 98% of the theoretical value, equivalent to ceramic injection molding results.

Furthermore, in the work of Eickhoff et al. [42], different combinations of LDPE, high-density polyethylene (HDPE), and wax were tried to make Ti6Al4V parts by FFF. It has been revealed that the mechanical and rheological properties may be modified for most of these formulations, which utilize varying grades of polyethylene (PE) as the primary component. Nevertheless, PP is a better candidate than PE as the backbone for metals with low sintering temperatures since PP degrades at lower temperatures than PE.

Aluminium (Al) and its alloys offer several benefits as superior materials in sophisticated and high-tech applications. Increasing demands for high thermal and electrical conductivity [43], low component weight [44], and outstanding specific mechanical properties [45] in the automotive, aerospace, electronics, and military industries have resulted in a surge in the use of Al products [46]. Nevertheless, processing Al powder by SDS is not very common, possibly due to difficulties encountered during the sintering process. One of the challenges is that the sintering temperature of Al is relatively near to the degradation temperature of most polymers. However, certain researchers have attained tremendous achievements. Several studies have been done on the MIM of Al and its alloys [47]. For example, Abdoos et al. [48] investigated the rheological behavior of Al feedstocks for injection molding. The binder system contains PP, paraffin wax (PW), and stearic acid (SA) in various percentages. The results showed that the proper binder system is 60, 35, and 5 vol.% of PW, PP, and SA, respectively.

Furthermore, the binder with extra PW (65 vol.%) had an unstable flow behavior. The main challenge to directly applying MIM Al feedstocks to FFF is that the high wax content makes the filaments unprintable. Therefore, new binder formulations are needed. One way to tailor the formulations is to select an appropriate polyolefin backbone, which was one of the aims of this investigation.

Although polyolefins have been used for the backbones in FFF binder systems for metals [32], there is no investigation to analyze the effects of the type of backbone on the feedstock properties by using a wax and a higher molecular weight PP (i.e., PP polymer vs. PP_wax_) and grafting. There is one study where a MIM Al feedstock was processed in a screw-based material extrusion additive manufacturing [49], but to our knowledge, there is no study on Al feedstock used in the FFF process in the literature. Additionally, some research has been done on the use of grafted backbones in which a beneficial increase in the polymer-powder interaction was observed [50]. For this reason, grafted polyolefins have been considered in this paper. Therefore, in this study, the rheological behavior, and mechanical and thermal properties of Al feedstocks for FFF were investigated using different PP as backbones to obtain a feedstock that can be reliably used to fabricate specimens in a low-cost FFF printer.

## 2. Materials and Experimental Methods

### 2.1. Materials

This study used an Al alloy powder with spherical particles and d_90_ < 32 µm to produce FFF feedstocks. The powder was supplied from TLS Technik Spezialpulver KG (Bitterfeld-Wolfen, Germany). It was referred to as AlSi1—three multi-component binder systems with a fixed soluble component and different PP grades as the backbone were utilized. The different grades of modified and unmodified PP consisted of (i) general application PP (Borealis AG, Vienna, Austria) with a density of 0.95 g/cm^3^, (ii) grafted-maleic anhydride-PP (PPMA) (BYK-Chemie GmbH, Wesel, Germany) with the density of 0.935 g/cm^3^ and, (iii) grafted-maleic anhydride-PP_wax_ (PPMA_wax_) (Clariant AG, Oberhausen, Germany) with the density of 0.93 g/cm^3^. A thermoplastic elastomer (TPE) (KRAIBURG TPE GmbH & Co. KG, Waldkraiburg, Germany) with a density of 0.944 g/cm^3^ and shore A hardness of 60 was used as the main binder component. According to the melt flow index (MFI) test at 190 °C with a load of 2.16 kg for three repetitions, the MFI of PP and PPMA were 2.27 ± 0.02 g/10 min and 6.37 ± 0.02 g/10 min, respectively. The viscosity of PPMA_wax_ is given by the manufacturer as 0.8–1.4 Pa·s.

The binder and feedstock were mixed in an internal mixer with a chamber volume of 38 cm^3^ and counter-rotating roller rotors (Plasti-Corder PL2000, Brabender GmbH & Co. KG, Duisburg, Germany) at the temperature of 200 °C and a rotational speed of 60 rpm. The mixing was done in an air atmosphere for 45 min to ensure adequate dispersion of powder particles in the binder system, which can be indicated by a constant torque value at the end of blending. For comparison, all the pure materials were processed in the same condition for 45 min. After mixing, the feedstocks were cooled and granulated at room temperature in a cutting mill (SM200, Retsch GmbH, Haan, Germany). Compounded samples were prepared in the ratio where the TPE consists of more than 50 vol.% of the binder composition. High TPE content keeps the filament flexible. After solvent debinding minimizes the amount of polymer that needs to be removed before sintering, which speeds up the thermal debinding process, saving time and energy.

### 2.2. Thermal Characterization

The melting and cooling behavior for the binder systems and feedstocks was characterised using a differential scanning calorimeter (DSC) (Mettler Toledo GmbH, Greifensee, Switzerland) based on ISO 11357. Measurements (3 replicates for each binder and feedstock) were carried out in the temperature range from 25 to 250 °C with a heating rate of 10 K/min and a cooling rate of 20 K/min under nitrogen gas (gas flow rate: 50 mL/min). Melting temperature, crystallization temperature, and degree of crystallinity for binder systems were measured. The degree of crystallinity in each virgin polymer and binder system was calculated according to Equation (1) [51].

(1) $$X_{c}=\frac{\Delta H_{m}}{\Delta H_{m}^{0}*w}*100$$

where $\Delta H_{m}$ is the enthalpy of melting, $\Delta H_{c}$ is the enthalpy of crystallization, $\Delta H_{m}^{0}$ is the enthalpy of 100% crystalized PP, which is 207 J/g [52], and w is the weight fraction of PP in the samples.

For analyzing the thermal stability of binder systems and feedstocks, thermogravimetric analysis (TGA) (Mettler Toledo GmbH, Greifensee, Switzerland) was performed based on ISO 11358. The test was executed to explore the variation of sample weight in wt.% versus temperature in an inert (N_2_) atmosphere. One test was done for each sample, and all the samples were heated from room temperature to 800 °C with a heating rate of 10 K/min.

### 2.3. Rheological Measurements

It is also essential to evaluate the rheological characteristics of the feedstocks at high shear rates because the FFF process occurs in this range. Therefore, the high-pressure capillary rheometer Rheograph 2002 (GÖTTFERT Werkstoff-Prüfmaschinen GmbH, Buchen, Germany) was used to investigate the rheological behavior of the feedstocks. The apparent shear viscosity based on ISO 11443 was measured at 200 °C in the apparent shear rate range of 100–1000 s^−1^; using a die with a diameter and length of 1 and 30 mm, respectively. Three repetitions were done for all samples, and the mean values were reported.

To make specimens for the rotational rheology, disks were manufactured using the hydraulic vacuum press P200PV (COLLIN Lab & Pilot Solutions GmbH, Maitenbeth, Germany) with a pressure of 50 bar at 200 °C. The disks were produced with a diameter of 25 mm and a thickness of about 1 mm. The feedstock sample was preheated between 15 and 20 min before pressing, and the cooling time was fixed at 15 min.

The rheological properties of binder systems and feedstocks at low shear rates were measured using a rotational rheometer MCR 702 MultiDrive (Anton Paar GmbH, Graz, Austria). The experiments were carried out in parallel plate configuration at 200 °C in an inert (N_2_) atmosphere with a plate diameter of 25 mm. Strain sweeps were carried out in the strain range (γ) from 0.01 to 10% at a fixed frequency of 1 rad/s to determine the linear viscoelastic region in the samples. Therefore, the frequency sweep tests according to ISO 6721 with the angular frequency range from 0.1 to 500 rad/s for the binder systems and feedstocks were done with γ = 1% and γ = 0.01%, respectively. All the samples had the same recent thermal history. At least three replicates were performed on fresh samples. For better comparison, virgin grades of PP and TPE were also examined.

### 2.4. Microscopy and Infrared Spectroscopy

Feedstock micromorphology was analyzed using scanning electron microscopy (field emission gun SEM, Zeiss Leo 1525, Carl Zeiss Microscopy, Jena, Germany) using backscattered electrons (15 kV source voltages). The compression molded specimens were mechanically ground and polished with the final step using a suspension of 0.1 µm alumina particles.

The Fourier transform infrared (FTIR) is one of the essential measurements to understand the internal interactions between materials. The FTIR tests used a VERTEX 70v FT-IR spectrometer (Bruker Optics GmbH & Co. KG, Ettlingen, Germany) to analyze the interaction between polymers in the binders and the Al fillers.

### 2.5. Tensile Testing

Furthermore, filaments were made in the high-pressure capillary rheometer with a die of 1.75 mm diameter and 30 mm length. The filament-making was done at 200 °C and a constant shear rate of 200 s^−1^. In the next step, the tensile properties of the filaments were measured according to ISO527 using a Zwick Z001 machine with a 1 kN load cell from ZwickRoell GmbH & Co. KG (Ulm, Germany) at room temperature. The initial gauge length and displacement rate were 50 mm and 10 mm/min, respectively. The mechanical properties results were averaged over five samples of each composition.

### 2.6. FFF Printing Trials

FFF printing was done on a Duplicator i3 v2 (Wanhao, Jinhua, China) machine with a 0.6 mm nozzle diameter. The printed geometry was a rectangular prism with a honeycomb infill at 40% and the dimension of 20 mm × 20 mm × 4 mm. The specimens (3 parts for each feedstock) were printed at a nozzle temperature and a building platform temperature of 240 °C and 100 °C, respectively, to ensure proper extrusion and build platform adhesion. The printing speed was kept at a maximum of 10 mm/min, and the retraction was reduced significantly to avoid excessive shearing of the filament. Furthermore, a low printing speed results in a low volume flow, thus a lower force acting on the filament. Hence, a more stable printing process can be achieved.

## 3. Results and Discussion

### 3.1. Thermal Characterization

#### 3.1.1. DSC Analysis

**Figure 1:**
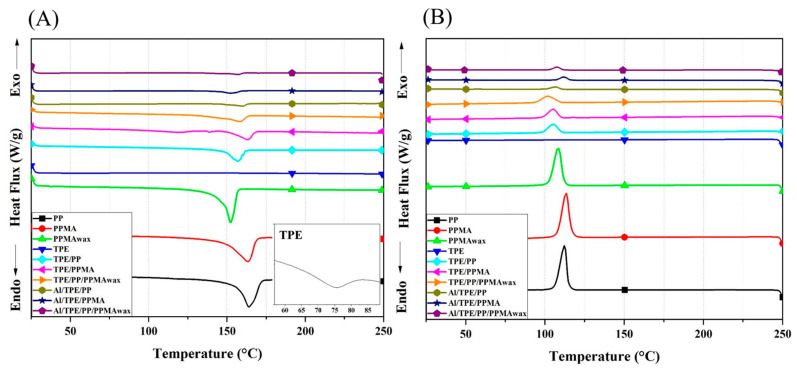
The average DSC curves of polymers, binder systems, and feedstocks, (**A**) second run of melting behavior, (**B**) cooling behavior of samples.

DSC was used to investigate the thermal properties and the degree of crystallinity. The thermographs of pure polymers, binder systems, and feedstocks are depicted in Figure 1, and the thermal properties are reported in Table 1. T_m_ is the melting temperature, T_c_ is the crystallization temperature, and X_c_ is the degree of crystallinity calculated using Equation (1). Processed PP shows the highest T_m,_ basically around 164 °C. Processed PPMA shows nearly the same T_m_ as PP. Furthermore, the difference between melting enthalpies of PP and PPMA is not significant. The primary polymer in the binder systems is TPE. Therefore, it would have a big influence on the thermal properties of binder systems. Pure TPE shows a small exothermic peak around 75 °C (Figure 1, inset), which is assumed to be the melting temperature of hard segments of the TPE molecular structure. It is assumed that the TPE is mainly composed of hard and soft segments, each of which provides different properties. The hard segment provides processability, while the soft segment provides elasticity and flexibility. Because the melting enthalpy of TPE is very small, we believe that the melting and crystalline properties of the blends based on TPE and PP would be mainly influenced by the type of PP in the system. After 45 min of processing, TPE at 200 °C the melting peak completely disappeared. By blending TPE, T_m,_ and the melting enthalpies were reduced.

As a comparison between TPE/PP and TPE/PP/PPMA_wax_, the reduction in T_m_ was bigger due to the presence of wax chains that can move and slide easily. Furthermore, the ease in chain movement would enhance the ability of the chain to move and stack on each other and increase the degree of crystallinity [53]. TPE/PPMA sample shows a big reduction in T_m_. This would indicate the presence of some chemical interaction among PPMA and TPE chains, which further hinders molecular movements and affects the melting temperature.

Regarding feedstocks, adding Al alloy powder to each binder system decreases T_m_, mostly due to the presence of metallic particles that impede the movement of the polymer chain. The presence of Al particles also reduces the melting enthalpy compared to the polymeric binder system.

Regarding the crystallization behavior of samples (Figure 1B), PPMA shows the highest T_c_, around 114 °C, resulting from easily packing of PPMA chains and forming a crystalline structure. Adding TPE to PP in binder systems reduces the crystallization temperature, meaning PP chains need more energy reduction to fold on each other and form a crystalline structure. In comparison to TPE/PP binder system, TEP/PP/PPMA_wax_ has a higher T_c_, which is mainly by the addition of low molecular weight wax to the system that increases chains movement and makes it easier for chains to fold on each other and make crystalline structures. The same behavior is also observed in TPE/PPMA binder system.

Regarding the degree of crystallinity, by blending TPE with different types of PP, the crystallinity in binder systems reduces significantly. This is mainly due to the higher content of non-crystalline TPE. Furthermore, by adding PPMA_wax_ to the TPE/PP binder system, although the content of PP was lower, a higher degree of crystallinity is observed, which is also due to a higher growth rate in this system because of the shorter wax chains [54].

In feedstocks, by adding Al alloy particles, the T_c_ for all the samples increased. It can result from the solid metallic particles, which act as a nucleating agent and further would increase the degree of crystallinity. As reported in Table 1, the crystallinity of Al/TPE/PPMA and Al/TPE/PP/PPMA_wax_ increased. However, in Al/TPE/PP system, a reduction in X_c_ was observed, which should be investigated further since no clear explanation is available. The degree of crystallinity for feedstocks would directly affect mechanical strength, which is addressed in the following sections.

#### 3.1.2. TGA Analysis

**Figure 2:**
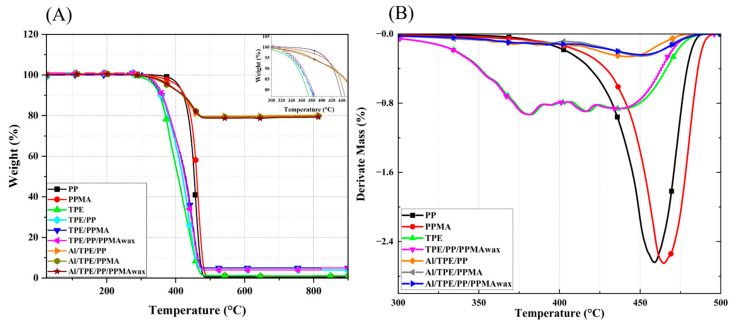
(**A**) TGA representative curve, and (**B**) dTGA curves of processed polymers, binder systems, and feedstocks.

Thermal degradation of the polymeric binder system is important in the FFF process of sensitive alloys, particularly Al, because according to [55], the beginning stages of sintering for Al alloys happen at temperatures around 550 °C and most binder polymers degrade up to these temperatures. TGA was used to investigate the degradation further, and the results are presented in Figure 2.

Processed PP has a thermal degradation onset temperature of around 340 °C. PPMA has a lower onset of thermal degradation (around 310 °C), probably due to 1 wt.% maleic anhydride modification in this material. The rate of thermal degradation of the polyolefin backbone can be considered the same. The degradation behavior of binder systems is mainly the same as TPE, with the onset of degradation temperature around 270 °C (the insert of Figure 2). The decrement in degradation temperature is beneficial to decreasing defects and contamination during thermal debinding and sintering.

Furthermore, the degradation rate of processed TPE and binder systems is lower than that of polyolefins. Processed PP and PPMA show an abrupt weight loss in a limited temperature window. By adding TPE to PP and PPMA, the rate of degradation reduces a little bit, and the rate becomes more moderate. Furthermore, the degradation of all binder systems and polymers terminates at around 500 °C. In conclusion, all the binder systems showed good thermal degradation behavior, which is essential for the SDS of Al feedstocks.

To further investigate the effect of the presence of metal particles on the binder system, TGA analyses were also conducted on the feedstocks. The onset of degradation for all feedstocks is around 300 °C. All samples (binder system and feedstock) containing PPMA showed lower thermal degradation temperatures. The degradation rate of feedstocks is smaller than that of binder systems, which is attributed to the presence of Al alloy particles that hinder thermal degradation and slow down the degradation process. Also, in the work of Bek et al. [56], MA chemically reacts with Al particles, which could be why the degradation is slowed down. To further investigate the thermal degradation of samples, dTGA data are depicted in Figure 2B. All the binder systems have the same degradation behavior as TPE. A two-step degradation peak is attributed to the degradation of each soft and hard segment present in TPE. We believe that due to the higher content of TPE in the binder system, the degradation behavior is more affected by the presence of TPE. However, the feedstocks show completely different behavior. The degradation happens in a one-step and at nearly the same temperature as PP and PPMA. Feedstocks degraded in a smaller temperature window which shows the suitability of these binder systems in the SDS process of Al.

### 3.2. Rheological Characterization

#### 3.2.1. High-Pressure Capillary Rheometer

Analyzing the viscosity is very important for FFF feedstocks because it is impossible to print feedstocks with very high viscosity [57]. On the other hand, making printable filaments using low-viscosity feedstocks is impossible since low-viscosity components (mainly waxes) tend to make very fragile filaments, which break during the extrusion process at the printing head.

**Figure 3:**
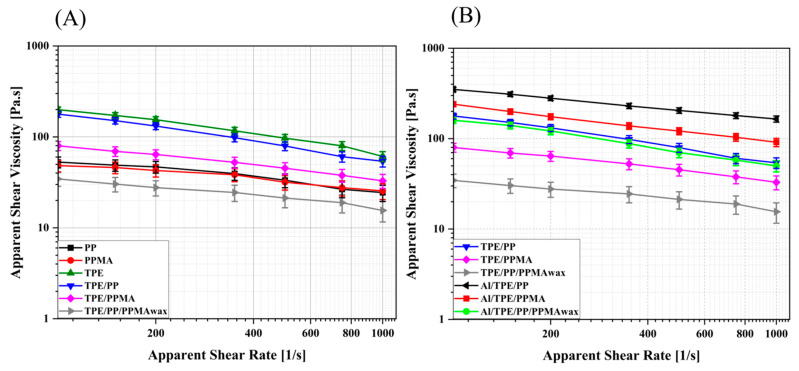
Average HPCR results, (**A**) binder systems and components, (**B**) binder systems and feedstocks.

A high-pressure capillary rheometer (HPCR) followed the flow characteristic of binder systems and feedstocks because, in FFF and PIM processes, the material is forced to flow through a narrow nozzle at high shear rates [58]. The average HPCR results of pure polymer and binder systems are depicted in Figure 3A. All samples show shear-thinning behavior, and the processed TPE shows the highest viscosity among the other polymers. Processed PP and PPMA show nearly the same flow properties and lower viscosity than TPE due to the presence of more free volume, making it easier for polymer chains to slide on each other even at high shear rates. As can be observed in Figure 3, the shear viscosity of TPE/PP is roughly the same as TPE, and TPE/PPMA has an even lower viscosity, which shows that at high shear rates, the binder systems flow more easily than the single binder ingredients. TPE/PP/PPMA_wax_ shows relatively low viscosity among all samples. PPMA_wax_ has a very low viscosity, and its viscosity was not measurable in a capillary rheometer at 200 °C.

The shear viscosity of the feedstocks is depicted in Figure 3B. By adding 55 vol.% Al alloy particles to binder systems, an increment in shear viscosity in comparison to the binder systems is seen, on average 2, 3, and 4.7 times for TPE/PP, TPE/PPMA, and TPE/PP/PPMA_wax_, respectively. The lower viscosity in Al/TPE/PP/PPMA_wax_ is due to the presence of the wax. However, the increment in viscosity in samples containing maleic anhydride-modified PP is higher, which can indicate some chemical interactions between Al alloy particles and maleic anhydride groups on the PP backbone. Therefore, we further analyzed the probable interactions with Fourier transform infrared spectroscopy (FTIR).

#### 3.2.2. Rotational Rheology

**Figure 4:**
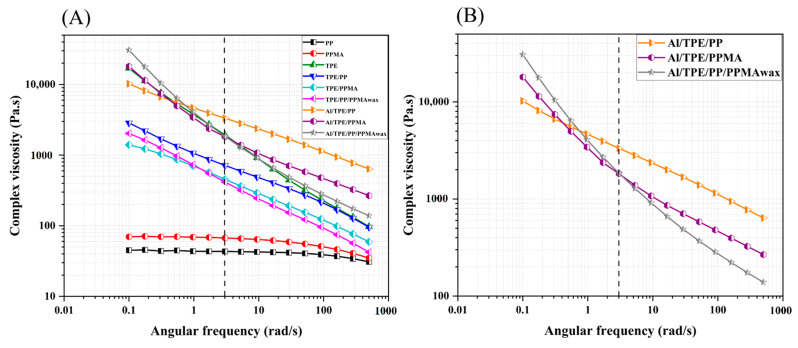
The mean values of complex viscosity, (**A**) processed polymers and binder systems, (**B**) feedstocks.

**Figure 5:**
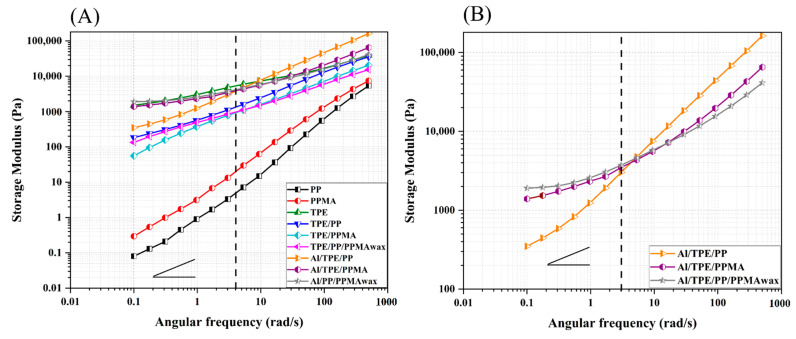
The mean values of storage modulus, (**A**) processed polymers and binder systems, (**B**) feedstocks.

Investigating the rheological property with rotational rheology is prominent in investigating the structure and interactions at lower frequencies or the flow behavior and processability at higher frequencies. To gain a deeper understanding of the interactions and flow behavior, rotational analysis was done on processed polymers, binder systems, and feedstocks, and the results are depicted in Figure 4 and Figure 5. Processed PP and PPMA show a plateau at lower angular frequencies followed by shear-thinning behavior at higher frequencies. In the range of rheological measurement, PPMA has a higher complex viscosity. PP and PPMA show the typical flow behavior of uncrosslinked thermoplastics (the rheological behavior of pure materials can be seen in the Appendix A—Figure A1). However, processed TPE shows a different behavior. There is no plateau; only shear thinning is observed in the measured angular frequency range.

In the binder systems, similar flow behavior is observed. At all frequencies, all the binder systems have a flow behavior similar to pure TPE but with lower viscosity values compared to TPE and a smaller shear-thinning slope. At all frequencies, TPE/PP shows a higher viscosity. At the medium to high frequencies, TPE/PPMA has a viscosity between TPE/PP and TPE/PP/PPMA_wax_. However, TPE/PP/PPMA_wax_ shows higher viscosity than TPE/PPMA at low frequencies. This is mainly due to the lower viscosity of PPMA_wax_.

In the case of feedstocks, the complex viscosities of samples are higher than binder systems, mainly at low frequencies (or shear rates) due to the presence of a high amount of metallic powder. This behavior is because, at low frequencies, the particles are more prone to make strong networks. In highly-filled composites, the particle-particle interactions have the main effect on the viscoelastic properties. The viscosity upturn in Al/TPE/PPMA and Al/TPE/PP/PPMA_wax_ indicates network formation in the sample, which will be confirmed with scanning electron microscopy analysis (SEM) (see Section 3.3). Furthermore, in the work of Bek et al. [56], it is reported that there is a chemical interaction between polymer chains and Al particles. A higher binding energy was observed in modified polymers, which means stronger adhesion of particles to the polymer backbone, which would hinder the polymer chain movements and lead to an increment in viscosity. Therefore, in the next sections, possible interactions in our system will be analyzed by FTIR.

At low frequencies, the difference between the complex viscosity of each feedstock and its binder system is bigger. This difference is constant for TPE/PP sample, but for other systems, the difference decreases with the frequency, which could be related to the presence of agglomerates in Al/TPE/PP. On the contrary, when particles become wet with the appropriate binder system, the particle-polymer interactions increase. This phenomenon is called network formation, which leads to viscosity upturn at lower frequencies. Furthermore, at high frequencies, there is a network breakup, which here is more pronounced in Al/TPE/PP/PPMA_wax_ and resulted in more shear-thinning behavior than Al/TPE/PP and Al/TPE/PPMA.

**Figure A2:**
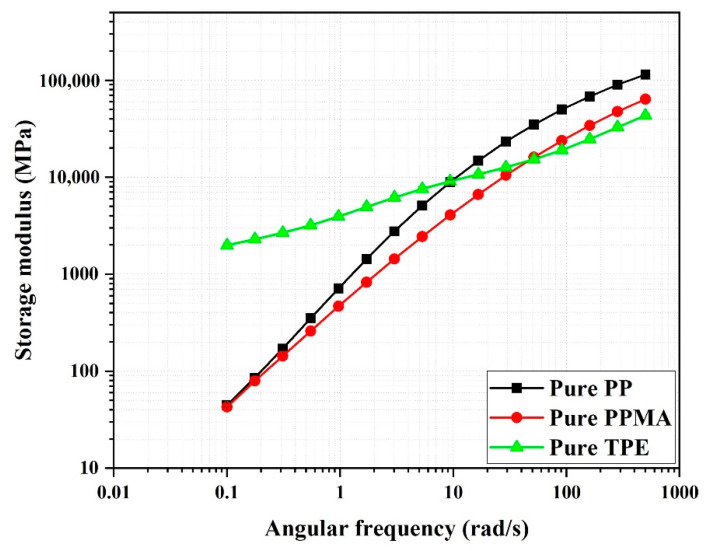
The storage modulus of the pure binder system components.

Roshchupkin et al. [59] reviewed the main ideas for the FFF of metals. They pointed out that the elasticity of melt and flexibility in solid and melt states are two crucial factors for binder systems in the FFF process. Hence, the dependency of storage modulus (G′) on frequency is a useful parameter to evaluate the elastic behavior of polymer melts. Among all processed samples, PP shows the lowest elasticity. The highest elasticity is observed in TPE due to its quasi-elastomeric nature that arises due to its non-covalent interactions between the soft and hard segments (Figure A2 in Appendix A). Furthermore, at low frequencies, the soft segments of TPE would not let G′ to decrease very much, and even at lower frequencies (ω < 0.1 rad/s), the flow behavior is like ideal elastic, where G′ is independent of frequency.

About the binder systems, the elastic behavior at every frequency is mainly similar to processed TPE. PP and PPMA show terminal behavior, while adding TPE to them, non-terminal behavior is observed, and the curves are shifted to a higher storage modulus. Among all the binder systems, TPE/PP shows the highest G′ at almost all frequencies. TPE/PPMA and TPE/PP/PPMA_wax_ have almost the same dependency of elasticity to frequency, except for some deviation at low frequencies in TPE/PP/PPMA_wax_ which further decreases the slope or the dependency of elasticity to frequency. Again, this behavior can be attributed to the presence of PPMA_wax_.

By adding particles, the storage modulus increased, and the behavior of feedstocks is nearly the same as processed TPE, except for Al/TPE/PP sample. More particle-polymer interactions, which result from better particle dispersion and increment in adhesion, have an actual effect on the value of G′ (Figure 5). The independency of the storage modulus of frequency, mainly at low frequencies (ω < 1 rad/s), is another indicator of network formation. This is further investigated by SEM results of the feedstocks based on TPE and various PP. The absorption of polymer chains on particles is more pronounced in Al/TPE/PPMA and Al/TPE/PP/PPMA_wax_ feedstocks, and based on FTIR results, it is believed that it may be due to more chemical bonding between particles and grafted polymer chains. Similar results have been reported by Bek et al. [56] and Cano Cano et al. [50]. Based on rheological results, among these feedstocks, TPE/PPMA and TPE/PP/PPMA_wax_ could be better in the FFF process. However, further investigation of the mechanical properties of feedstock filaments and their printability is needed to confirm this hypothesis.

### 3.3. Morphological Analysis

**Figure 6:**
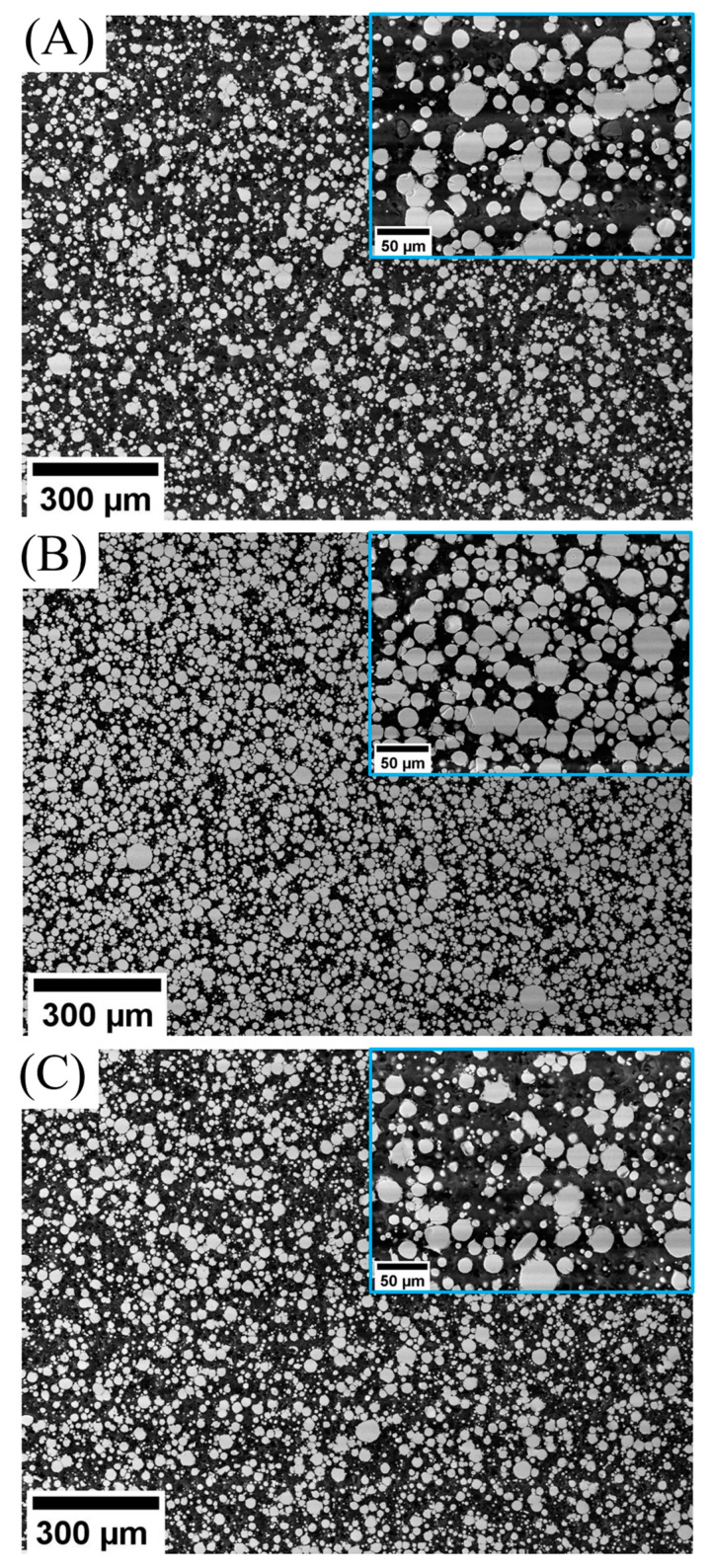
SEM images of (**A**) Al/TPE/PP, (**B**) Al/TPE/PPMA, and (**C**) Al/TPE/PP/PPMA_wax_.

To further investigate the micromorphology developed after making the feedstocks and the probable presence of agglomerates and networks in the sample, scanning electron analysis was performed on the compressed molded feedstock samples, and some of the results are depicted in Figure 6. The black and white regions are polymer and Al alloy particles, respectively.

A uniform distribution of the polymer phase is essential in the SDS process because if the polymer phase accumulates in one region, cracks will appear during debinding. As discussed in the rheology section, it was hypothesized that in Al/TPE/PP, agglomeration would be observed and, in both Al/TPE/PPMA and Al/TPE/PP/PPMA_wax_, a better dispersion should be observed (due to the upturn in viscosity and the plateau region in storage modulus results). In Figure 6A, an obvious agglomeration can be detected in Al/TPE/PP, where Al particles are directly in contact with each other (inset). Also, the particles are distributed more unevenly in the Al/TPE/PP sample, which can cause some problems like voids and cracking during debinding due to binder-rich zones. Furthermore, even if debinding could be accomplished, the sintering process might cause the development of large pores, leading to poor mechanical properties. In Al/TPE/PPMA sample (Figure 6B) a proper distribution of the Al particles can be observed. The surface detachment is not observed. As shown in Figure 6B (inset), there is a thin polymer layer around Al particles, which confirms the polymer adhesion to the metal particles that increase the viscosity of feedstocks. The Al/TPE/PP/PPMA_wax_ sample has an intermediate dispersive morphology. Even though there are still binder-rich regions, their sizes are smaller than the zones in the Al/TPE/PP sample, and the phase distribution is not uniform. Furthermore, agglomerates can be seen in the sample (Figure 6C).

### 3.4. Fourier-Transform Infrared Spectroscopy (FTIR)

**Figure 7:**
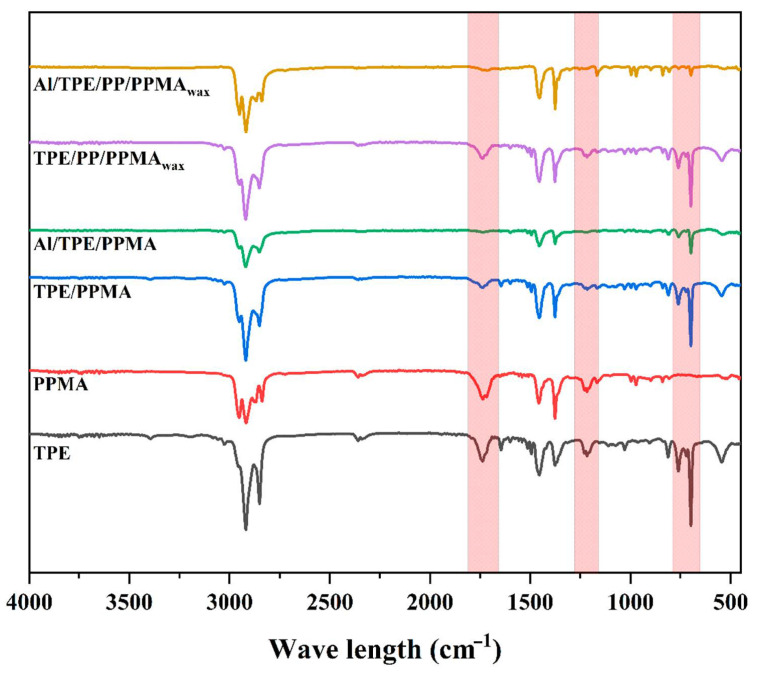
The representative FTIR spectrum of processed polymers, binder systems, and feedstocks.

To further investigate the probable chemical interactions in the binder system and feedstocks, Fourier-transform infrared spectrum (FTIR) was used, and some of the results are depicted in Figure 7. The characteristic peaks for TPE are its fingerprints at 690 and 760 cm^−1^, with peaks around 1730 cm^−1^ attributed to C=O groups. For PPMA also the peak at 1730 cm^−1^ is for C=O and 1210 cm^−1^ for C-O in maleic anhydride. By blending TPE with PPMA, no new peak appeared, but obviously, the intensities of some peaks were reduced. By the reduction in peaks at 1210 and 1730 cm^−1^, some chemical interactions can be assumed, which reduced the amount of C-O and C=O groups in the system. For both binder systems, the peak at 1730 cm^−1^ shifts to 1737 cm^−1^, which would be an actual reason for probable chemical interactions between TPE and PPMA. Different researchers also investigated the probable interaction of the polymer with metallic particles using FTIR. Bek et al. [56] analyzed the AlSi10 Mg surface, and they observed a high amount of Al_2_O_3_ compared to the elemental Al, which indicates high oxidation of the surface. Oxides on the metal alloy surface can interact with the components of the binder system, particularly with the maleic anhydride (MA). It is reasonable to assume that the MA ring-opened and reacted with the Al filler. A peak shift (associated with Al-O-H vibrations of filler) was observed, indicating the binding of the Al-O surface group located on Al filler material to the binder system and the formation of a covalent bond between Al filler and PPMA.

After preparing the feedstocks, the intensity of fingerprints of TPE lowered, which is due to less TPE in the system, compared to Al particles. However, the characteristic peaks at 1210 and 1730 cm^−1^ almost vanished, indicating chemical interactions between the binder system and Al particles. These results confirm our previous observations in the thermal and rheological sections.

### 3.5. Mechanical Properties and Printability

**Figure 8:**
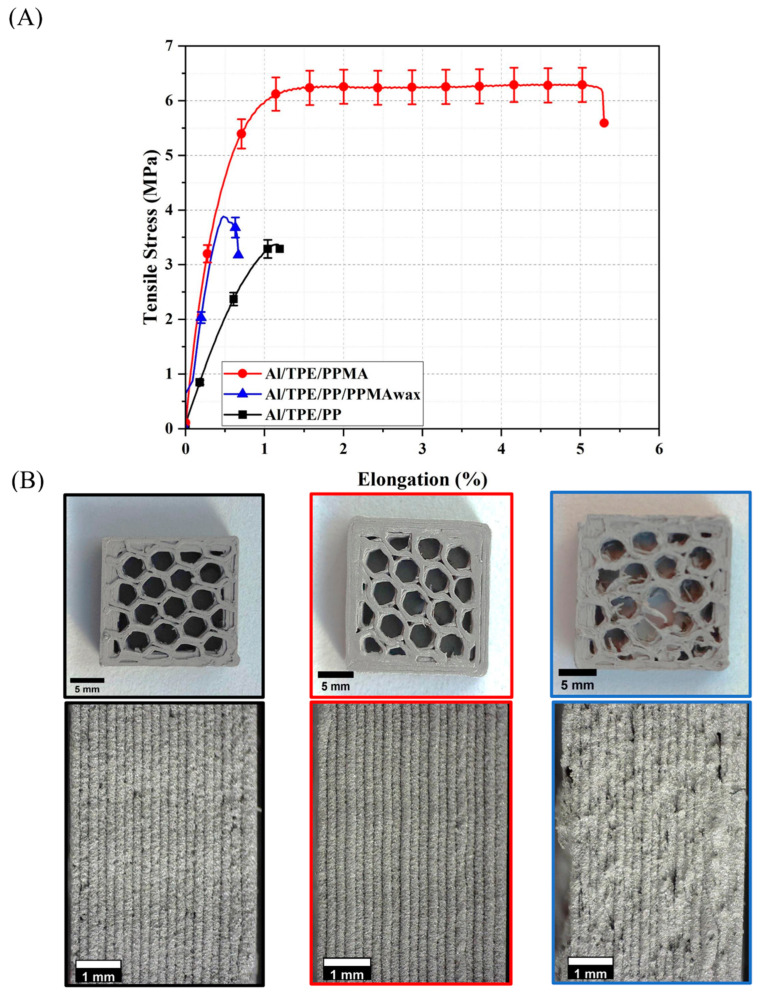
(**A**) The average tensile properties of the filaments, (**B**) pictures of as-printed samples from left to right: Al/TPE/PP, Al/TPE/PPMA, and AL/TPE/PP/PPMA_wax_.

To investigate the mechanical properties of the filaments and further produce green parts by 3D printing, the tensile test on filaments and their printability was conducted, and the results are depicted in Figure 8. Some of the printed parts with mean values of the tensile test results can be seen in Figure 8. The mechanical strength of the filaments is crucial because the green part is produced using filaments, and low strength and very brittle material can cause problems during the printing and handling of green parts. Figure 8 shows Al/TPE/PP and Al/TPE/PP/PPMA_wax_ show brittle behavior with low tensile strength around 3.4 and 3.9 MPa, respectively. However, the Al/TPE/PPMA sample shows a 1.6 to 1.8 times improvement compared to the two other feedstocks. This would further justify the suitability of the TPE/PPMA binder system in the FFF process.

The printed parts can be seen in Figure 8B. For Al/TPE/PP and Al/TPE/PPMA feedstocks, better printability is observed. However, big defects and irregularities are observed in the Al/TPE/PP/PPMAwax printed sample. The lower viscosity at the high printing temperature of 240 °C and the lowest elongation at break in the Al/TPE/PP/PPMAwax feedstock filament makes the extrusion process more unreliable. Hence, the part had the lowest quality (Figure 8B).

## 4. Conclusions

This study investigated the thermal, mechanical, and rheological properties of the Al feedstock in the FFF process. Different PP-based backbones were used to analyze the impact of backbones in the suitable feedstocks for FFF. According to the DSC results, it can be concluded that the sample with unmodified PP as the backbone has a lower crystallinity value compared to the samples which have maleic anhydride grafted to the PP in the binder systems and feedstocks. In the TGA results, it has been found that samples containing PPMA show lower degradation temperature, and compared to the PP, PPMA starts to degrade at lower temperatures (around 310 °C) because of the maleic anhydride. From the rheological point of view, all samples show shear-thinning behavior, which is appropriate for the FFF process. In addition, chemical interactions between Al alloy particles and maleic anhydride groups on the PP backbone led to higher changes in viscosity for samples with maleic anhydride-grafted PP. Also, FTIR results showed that there is a possibility of chemical bonding between oxides on the Al particle surface with the maleic anhydride in the binder system. Therefore, TPE/PPMA and TPE/PP/PPMAwax are preferable to the TPE/PP binder system in the FFF process regarding the rheological behavior. However, the filaments of the feedstocks with TPE/PP/PPMAwax as the binder system were brittle, with the lowest elongation at break, as seen in tensile testing results.

Further investigations should be performed to investigate the debinding and sintering behavior of the feedstocks to determine the suitability of the prepared feedstocks to obtain Al alloy specimens. However, this is out of the scope of this investigation.

## Figures and Tables

**Table 1:** The average thermal properties of the binder systems and components.

Sample	Heating		Cooling		
	T_m_ in °C	∆H_m_ in J/g	T_c_ [°C]	∆H_c_ [J/g]	X_c_ [%]
PP	164.30 ± 1.01	65.52 ± 0.15	112.20 ± 0.78	67.02 ± 0.64	31.65 ± 0.57
PPMA	163.24 ± 0.57	76.58 ± 0.96	113.60 ± 0.43	71.07 ± 0.41	37.00 ± 0.68
TPE	75.10 ± 0.89	0.60 ± 0.12	-	-	-
TPE/PP	161.11 ± 1.51	21.39 ± 0.51	105.15 ± 1.64	22.88 ± 0.65	2.03 ± 0.08
TPE/PPMA	156.29 ± 1.25	21.62 ± 0.67	104.65 ± 1.34	25.45 ± 0.31	5.28 ± 0.01
TPE/PP/PPMA_wax_	156.41 ± 1.78	22.19 ± 0.75	101.50 ± 1.86	23.97 ± 0.80	2.43 ± 0.10
Al/TPE/PP	160.60 ± 1.84	5.72 ± 0.68	106.62 ± 2.06	5.83 ± 0.26	0.76 ± 0.05
Al/TPE/PPMA	153.00 ± 1.68	5.56 ± 0.89	111.77 ± 1.45	6.72 ± 0.13	8.00 ± 0.15
Al/TPE/PP/PPMA_wax_	156.20 ± 1.96	5.02 ± 0.98	107.36 ± 2.64	6.67 ± 0.68	11.38 ± 0.02

**Figure A1:**
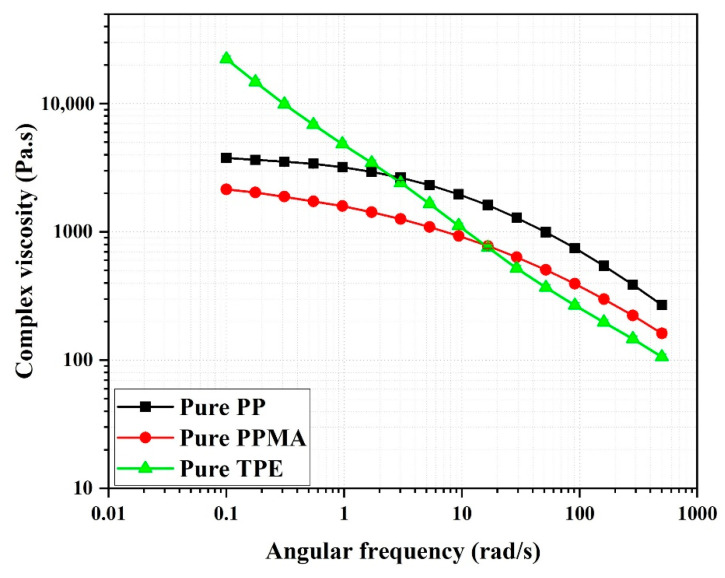
The complex viscosity of the pure binder system components.

## Data Availability

The data presented in this study are available on request from the corresponding author. The data are not publicly available due to commercial reasons.

## References

[1] Hadian A., Fricke M., Liersch A., Clemens F. (2022). Material extrusion additive manufacturing of zirconia parts using powder injection molding feedstock compositions. Addit. Manuf..

[2] Zhao Z., Liu R., Chen J., Xiong X. (2023). Additive manufacturing of cemented carbide using analogous powder injection molding feedstock. Int. J. Refract. Met. Hard Mater..

[3] Hwang I.-S., So T.-Y., Lee D.-H., Shin C.-S. (2023). Characterization of Mechanical Properties and Grain Size of Stainless Steel 316L via Metal Powder Injection Molding. Materials.

[4] Demers V., Turenne S., Scalzo O. (2015). Impact of binders on viscosity of low-pressure powder injection molded Inconel 718 superalloy. J. Mater Sci..

[5] Momeni V., Askari A., Allaei M.H., Zangi H. (2021). Investigating the Effect of Stearic Acid on the Mechanical, Rheological, and Microstructural Properties of AISI 4605 Feedstock for Metal Injection Molding Process. Trans. Indian Inst. Met..

[6] Fayyaz A., Muhamad N., Sulong A.B., Rajabi J., Wong Y.N. (2014). Fabrication of cemented tungsten carbide components by micro-powder injection moulding. J. Mater. Process. Technol..

[7] Naseer A., Ahmad F., Ali S., Haider W. (2022). Powder injection molded nano copper oxide grafted graphene reinforced copper matrix composites. Powder Technol..

[8] Attia U.M., Alcock J.R. (2012). Fabrication of hollow, 3D, micro-scale metallic structures by micro-powder injection moulding. J. Mater. Process. Technol..

[9] Momeni V., Alaei M.H., Askari A., Rahimi A.H., Nekouee K. (2020). Effect of the Fraction of Steel 4605 Powder in the Load in Injection Molding with the Use of a Polymer-Based Binder. Met. Sci. Heat Treat.

[10] Sidambe A.T., Figueroa I.A., Hamilton H., Todd I. (2012). Metal injection moulding of CP-Ti components for biomedical applications. J. Mater. Process. Technol..

[11] Ghasemi-Mobarakeh L., Cano S., Momeni V., Liu D., Duretek I., Riess G., Kukla C., Holzer C. (2022). Effect of Increased Powder-Binder Adhesion by Backbone Grafting on the Properties of Feedstocks for Ceramic Injection Molding. Polymers.

[12] Shahrubudin N., Lee T.C., Ramlan R. (2019). An Overview on 3D Printing Technology: Technological, Materials, and Applications. Procedia Manuf..

[13] Vozárová M., Neubauer E., Bača Ľ., Kitzmantel M., Feranc J., Trembošová V., Peciar P., Kritikos M., Orlovská M., Janek M. (2023). Preparation of fully dense boron carbide ceramics by Fused Filament Fabrication (FFF). J. Eur. Ceram. Soc..

[14] Momeni V., Zangi H., Allaei M.H. (2020). Effect of polypropylene as the backbone of MIM feedstock on the micro-structural phase constituents, mechanical and rheological properties of 4605 low alloy steel compacts. Powder Metall..

[15] Gibson M.A., Mykulowycz N.M., Shim J., Fontana R.R., Schmitt P., Roberts A., Ketkaew J., Shao L., Chen W., Bordeenithikasem P. (2018). 3D printing metals like thermoplastics: Fused filament fabrication of metallic glasses. Mater. Today.

[16] Momeni V., Hufnagl M., Shahroodi Z., Gonzalez-Gutierrez J., Schuschnigg S., Kukla C., Holzer C. (2022). Research Progress on Low-Pressure Powder Injection Molding. Materials.

[17] Atatreh S., Alyammahi M.S., Vasilyan H., Alkindi T., Susantyoko R.A. (2023). Evaluation of the infill design on the tensile properties of metal parts produced by fused filament fabrication. Results Eng..

[18] (2023). Ultrafuse FFF, Metal—Ultrafuse FFF. https://www.ultrafusefff.com/product-category/metal/.

[19] (2023). Zetamix, Filaments—Zetamix. https://zetamix.fr/en/category/filaments-en.

[20] (2023). Green Polymer Additives, 3D Printing|Green Polymer Additives|Emery Oleochemicals. https://greenpolymeradditives.emeryoleo.com/3dprinting/.

[21] Lengauer W., Duretek I., Fürst M., Schwarz V., Gonzalez-Gutierrez J., Schuschnigg S., Kukla C., Kitzmantel M., Neubauer E., Lieberwirth C. (2019). Fabrication and properties of extrusion-based 3D-printed hardmetal and cermet components. Int. J. Refract. Met. Hard Mater..

[22] Masood S.H., Song W.Q. (2005). Thermal characteristics of a new metal/polymer material for FDM rapid prototyping process. Assem. Autom..

[23] Zhang Y., Poli L., Garratt E., Foster S., Roch A. (2020). Utilizing Fused Filament Fabrication for Printing Iron Cores for Electrical Devices. 3D Print. Addit. Manuf..

[24] Damon J., Dietrich S., Gorantla S., Popp U., Okolo B., Schulze V. (2019). Process porosity and mechanical performance of fused filament fabricated 316L stainless steel. RPJ.

[25] Liu B., Wang Y., Lin Z., Zhang T. (2020). Creating metal parts by Fused Deposition Modeling and Sintering. Mater. Lett..

[26] Zhang Y., Bai S., Riede M., Garratt E., Roch A. (2020). A comprehensive study on Fused Filament Fabrication of Ti-6Al-4V structures. Addit. Manuf..

[27] Singh P., Balla V.K., Tofangchi A., Atre S.V., Kate K.H. (2020). Printability studies of Ti-6Al-4V by metal fused filament fabrication (MF3). Int. J. Refract. Met. Hard Mater..

[28] Bose A., Schuh C.A., Tobia J.C., Tuncer N., Mykulowycz N.M., Preston A., Barbati A.C., Kernan B., Gibson M.A., Krause D. (2018). Traditional and additive manufacturing of a new Tungsten heavy alloy alternative. Int. J. Refract. Met. Hard Mater..

[29] Carreira P., Cerejo F., Alves N., Vieira M.T. (2020). In Search of the Optimal Conditions to Process Shape Memory Alloys (NiTi) Using Fused Filament Fabrication (FFF). Materials.

[30] Wolff M., Mesterknecht T., Bals A., Ebel T., Willumeit-Römer R. (2019). FFF of Mg-Alloys for Biomedical Application.

[31] Schaper J.G., Wolff M., Wiese B., Ebel T., Willumeit-Römer R. (2019). Powder metal injection moulding and heat treatment of AZ81 Mg alloy. J. Mater. Process. Technol..

[32] Wagner M.A., Hadian A., Sebastian T., Clemens F., Schweizer T., Rodriguez-Arbaizar M., Carreño-Morelli E., Spolenak R. (2022). Fused filament fabrication of stainless steel structures—From binder development to sintered properties. Addit. Manuf..

[33] Momeni V., Zangi H., Alaei M.H. (2022). Effect of thermal debinding and sintering parameters on the mechanical properties of 4605 MIM compact using the RSM. Adv. Mater. Process. Technol..

[34] Rolere S., Soupremanien U., Bohnke M., Dalmasso M., Delafosse C., Laucournet R. (2021). New insights on the porous network created during solvent debinding of powder injection-molded (PIM) parts, and its influence on the thermal debinding efficiency. J. Mater. Process. Technol..

[35] Suwanpreecha C., Manonukul A. (2022). A Review on Material Extrusion Additive Manufacturing of Metal and How It Compares with Metal Injection Moulding. Metals.

[36] Hausnerova B. (2017). Binder systems for powder injection molding: A review. Adv. Mater. Proc..

[37] Gökmen U., Türker M. (2017). An Analysis of Rheological Properties of Inconel 625 Superalloy Feedstocks Formulated with Backbone Binder Polypropylene System for Powder Injection Molding. Arch. Metall. Mater..

[38] Cano S., Gonzalez-Gutierrez J., Sapkota J., Spoerk M., Arbeiter F., Schuschnigg S., Holzer C., Kukla C. (2019). Additive manufacturing of zirconia parts by fused filament fabrication and solvent debinding: Selection of binder formulation. Addit. Manuf..

[39] Gorjan L., Galusca C., Marwah S., Sebastian T., Clemens F. (2020). Effect of stearic acid on rheological properties and printability of ethylene vinyl acetate based feedstocks for fused filament fabrication of alumina. Addit. Manuf..

[40] Sadaf M., Bragaglia M., Nanni F. (2021). A simple route for additive manufacturing of 316L stainless steel via Fused Filament Fabrication. J. Manuf. Process..

[41] Nötzel D., Hanemann T. (2020). New Feedstock System for Fused Filament Fabrication of Sintered Alumina Parts. Materials.

[42] Eickhoff R., Antusch S., Baumgärtner S., Nötzel D., Hanemann T. (2022). Feedstock Development for Material Extrusion-Based Printing of Ti6Al4V Parts. Materials.

[43] Tatar C., Özdemir N. (2010). Investigation of thermal conductivity and microstructure of the α-Al_2_O_3_ particulate reinforced aluminum composites (Al/Al_2_O_3_-MMC) by powder metallurgy method. Phys. B Condens. Matter.

[44] Gökçe A., Fındık F., Kurt A.O. (2011). Microstructural examination and properties of premixed Al–Cu–Mg powder metallurgy alloy. Mater. Charact..

[45] Akhlaghi F., Zare-Bidaki A. (2009). Influence of graphite content on the dry sliding and oil impregnated sliding wear behavior of Al 2024–graphite composites produced by in situ powder metallurgy method. Wear.

[46] Rahimian M., Ehsani N., Parvin N., Baharvandi H.R. (2009). The effect of particle size, sintering temperature and sintering time on the properties of Al–Al_2_O_3_ composites, made by powder metallurgy. J. Mater. Process. Technol..

[47] Liu Z.Y., Sercombe T.B., Schaffer G.B. (2008). Metal injection moulding of aluminium alloy 6061 with tin. Powder Metall..

[48] Abdoos H., Khorsand H., Yousefi A.A. (2014). Torque rheometry and rheological analysis of powder–polymer mixture for aluminum powder injection molding. Iran Polym. J..

[49] Dayam S., Tandon P., Priyadarshi S. (2022). Development of paste extrusion-based metal additive manufacturing process. Rapid Prototyp. J..

[50] Cano S., Gooneie A., Kukla C., Rieß G., Holzer C., Gonzalez-Gutierrez J. (2020). Modification of Interfacial Interactions in Ceramic-Polymer Nanocomposites by Grafting: Morphology and Properties for Powder Injection Molding and Additive Manufacturing. Appl. Sci..

[51] Tarani E., Arvanitidis I., Christofilos D., Bikiaris D.N., Chrissafis K., Vourlias G. (2023). Calculation of the degree of crystallinity of HDPE/GNPs nanocomposites by using various experimental techniques: A comparative study. J. Mater Sci..

[52] Wunderlich B. (1990). Thermal Analysis.

[53] Piorkowska E., Rutledge G.C. (2013). Handbook of Polymer Crystallization.

[54] Thomas S., Arif P.M., Gowd E.B., Kalarikkal N. (2018). Crystallization in Multiphase Polymer Systems.

[55] Martı J., Castro F. (2003). Liquid phase sintering of P/M aluminium alloys: Effect of processing conditions. J. Mater. Process. Technol..

[56] Bek M., Gonzalez-Gutierrez J., Kukla C., Črešnar K.P., Maroh B., Perše L.S. (2020). Rheological Behaviour of Highly Filled Materials for Injection Moulding and Additive Manufacturing: Effect of Particle Material and Loading. Appl. Sci..

[57] Hadian A., Koch L., Koberg P., Sarraf F., Liersch A., Sebastian T., Clemens F. (2021). Material extrusion based additive manufacturing of large zirconia structures using filaments with ethylene vinyl acetate based binder composition. Addit. Manuf..

[58] Strano M., Rane K., Vangosa F.B., Di Landro L. (2019). Extrusion of metal powder-polymer mixtures: Melt rheology and process stability. J. Mater. Process. Technol..

[59] Roshchupkin S., Kolesov A., Tarakhovskiy A., Tishchenko I. (2021). A brief review of main ideas of metal fused filament fabrication. Mater. Today Proc..

